# Human Viruses and Cancer

**DOI:** 10.3390/v6104047

**Published:** 2014-10-23

**Authors:** Abigail Morales-Sánchez, Ezequiel M. Fuentes-Pananá

**Affiliations:** 1 Unidad de Investigación en Virología y Cáncer, Hospital Infantil de México Federico Gómez. Dr. Márquez 162, Col. Doctores, C.P. 06720. México D.F., Mexico; E-Mail: abimor2002@yahoo.com.mx; 2 Programa de Doctorado en Ciencias Biomédicas, Facultad de Medicina, Universidad Nacional Autónoma de Mexico. Av. Universidad 3000, Col. Universidad Nacional Autónoma de México, C.U., C.P. 04510 México D.F., México

**Keywords:** infectious cancer, human oncogenic viruses, transforming mechanisms

## Abstract

The first human tumor virus was discovered in the middle of the last century by Anthony Epstein, Bert Achong and Yvonne Barr in African pediatric patients with Burkitt’s lymphoma. To date, seven viruses -EBV, KSHV, high-risk HPV, MCPV, HBV, HCV and HTLV1- have been consistently linked to different types of human cancer, and infections are estimated to account for up to 20% of all cancer cases worldwide. Viral oncogenic mechanisms generally include: generation of genomic instability, increase in the rate of cell proliferation, resistance to apoptosis, alterations in DNA repair mechanisms and cell polarity changes, which often coexist with evasion mechanisms of the antiviral immune response. Viral agents also indirectly contribute to the development of cancer mainly through immunosuppression or chronic inflammation, but also through chronic antigenic stimulation. There is also evidence that viruses can modulate the malignant properties of an established tumor. In the present work, causation criteria for viruses and cancer will be described, as well as the viral agents that comply with these criteria in human tumors, their epidemiological and biological characteristics, the molecular mechanisms by which they induce cellular transformation and their associated cancers.

## 1. Introduction: Historical and Epidemiological Aspects

The first observations about a possible infectious etiology of cancer arose at the beginning of the past century. Ellermann and Bang in 1908 and Rous in 1911 transmitted avian leukemias and sarcomas, respectively, through cell-free tumor extracts, suggesting a viral etiology [1,2,3]. About 50 years later, the first human tumor virus was discovered. Sir Anthony Epstein, Bert Achong and Yvonne Barr observed viral particles in cell cultures from equatorial African pediatric patients with Burkitt’s lymphoma; this virus was named Epstein Barr virus (EBV) in honor of their discoverers [4]. In the following years, a set of experimental evidence demonstrated that EBV was the causative agent of endemic Burkitt’s lymphoma and other neoplasias. Currently, there is clear evidence that several viruses are oncogenic to humans and the first century of tumor virology research has culminated with the Medicine Nobel Price granted to Harald zur Hausen for the discovery of HPV as the causative agent of cervical cancer [5,6]. To date, EBV, Kaposi’s sarcoma-associated herpesvirus (KSHV), human high-risk papillomaviruses (HPV), Merkel cell polyomavirus (MCPV), hepatitis B virus (HBV), hepatitis C virus (HCV) and Human T-cell Lymphotropic virus type 1 (HTLV1) have been classified as type 1 carcinogenic agents (the most strongly associated with human cancers) by the International Agency for Research on Cancer (IARC) (reviewed in [7]). It is estimated that infections are responsible for up to 15% of cancer cases worldwide and about 20% in developing countries [8]. With advent of new technologies allowing genetic identification, it is very likely that this numbers will continue to increase.

Virus-mediated oncogenesis results from the cooperation of multiple events, including different mechanisms bound to the viral life cycle. The knowledge derived from the study of tumor viruses has allowed the construction of a conceptual biological framework to understand not only cancers of infectious origin but also of almost any type of cancer. However, to change the traditional scientific thinking to accept the participation of infectious agents in cancer was difficult, mostly because the biological processes involved do not adjust to the causation dogmatic principles postulated by Koch [9] (Table 1). Koch original observations about the transmission of acute infectious agents are difficult to apply to cancer because of the multi-factorial nature of cancer and because tumorigenic viruses are generally present in a large part of the population without causing disease. Sir Austin Bradford Hill’s epidemiologic causation criteria, which were originally proposed to establish the causation between smoking and lung cancer, are more suitable as a base to infer a causative relationship between a viral infection and cancer (Table 1) [10].

It is also accepted that none of the Bradford Hill’s criteria could by itself conclude causation, neither it is necessary to comply with all of them to accept the virus-cancer association. For example, the geographic distribution of endemic Burkitt’s lymphoma (equatorial Africa) does not coincide with the world distribution of EBV. However, we know today that malaria, endemic to this region, is a critical co-factor to develop Burkitt’s lymphoma (reviewed in [11]). The Bradford Hill criteria applied to virus and cancer associations consider that causation is established if the virus is present in the tumor cells and not in the surrounding healthy tissue and if there exists plausibility and coherence between infection and cancer. For example, EBV resides in B-lymphocytes that reactivate in the epithelium of the upper digestive tract and EBV has been associated to B-cell lymphomas and carcinomas in tongue, nasopharynx and stomach. Also, transgenic animals that express EBV latent proteins develop neoplasias [12]. These combined data grant a minimal context for biologic plausibility and coherence required by the Bradford Hill criteria.

**Table 1:** Koch and Bradford Hill’s postulates for causative relations.

Henle Koch’s Postulates [9]	Bradford Hill’s Causative Principles [10]
The pathogen agent must be present in sick population and absent in healthy population. The agent must not appear randomly in another disease. The agent can be isolated and cultured from a diseased organism and should cause disease when introduced into a healthy organism. The agent isolated from the new host should be identical to the original causative agent.	Strength of the association. The agent must be more common in cases than in healthy controls. Consistency. Different researchers must corroborate the association. Specificity. The disease must coexist with the agent in the same space, preferably over other associations of the same agent and another disease. Temporality. Exposure to the agent must predict the appearance of the disease. Gradient. A higher exposure must correlate with a higher probability to develop the disease. Plausibility. The association must be founded on the known biological aspects of the causative agent. Coherence. The association must be based on the known aspects of the disease. Experimental. Controlled conditions must reproduced and coincide with the disease and the blockage of biological mechanisms involved must reduce or prevent its appearance. Analogy. Agents with similar mechanisms must be associated to similar diseases.

Arguable, the most powerful tool to indicate direct association is the viral monoclonal analysis in the tumor; the presence of a specific viral variant or viral quasispecie in all tumor cells indicates that the event of infection preceded the malignant cell transformation. This strongly supports that the virus was part of the initial genetic lesion that allowed the appearance of the cancerous clone, satisfying the Bradford Hill criteria for temporality.

## 2. General Principles of Viral Oncogenic Mechanisms

Oncogenic viruses generally maintain chronic infections in which there is not or little production of viral particles, and that last for the whole life of the infected individual. These mechanisms of viral persistency and/or latency are biologically compatible with the carcinogenic process, because they avoid cell death most common in acute lytic infections, while maintaining the infectious agent hidden from the immune system. Viral persistence in the host is achieved by integrating the viral genome into the cell genome or by expressing viral proteins that equally segregate the viral genome into daughter cells during cell partitioning. Both mechanisms ensure that the virus is not lost during cellular replication. Viral persistence is usually characterized by expression of proteins that control cell death and proliferation; in this manner, oncogenic viruses nurture infection of a controlled number of cells establishing a balance between virus and host, preserving the integrity of both. Cell transformation is probably not an evolutionary viral strategy, but rather a biological accident that rarely occurs in the virus-host interaction. Cancer leads to the death of the host, and thus, it also represents the end of the virus. The existence of viral oncogenes is explained as part of the viral persistence mechanisms, which only under altered conditions may lead to cancer. All virus-associated tumors result from the cooperation of various events, involving more than persistent infection and viral transformation mechanisms. Additional oncogenic hits are necessary for full-blown transformation. The occurrence of mutations impairing expression and function of viral and/or cellular oncogenes is necessary in the carcinogenic process, in line with that, an increased mutation rate of infected over normal cells is frequently observed (reviewed in [13,14]). In this scenario, latently infected cells by oncogenic viruses might be more susceptible targets of additional oncogenic hits; e.g., due to smoking, a diet scarce in fruits and vegetables or/and increased exposure to environmental oncogenic agents. All these insults, plus the host genetic component driving inflammatory responses triggered by the infection itself result in cell transformation and cancer development.

### 2.1. Direct and Indirect Viral Carcinogenesis

**Figure 1:**
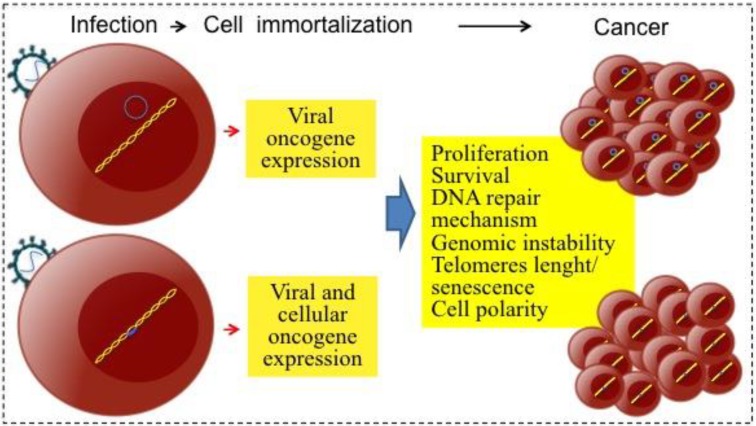
Direct mechanisms of viral carcinogenesis. After infecting target cells, tumor viruses are persistently maintained as genetic elements; viral genomes can form episomes (upper panel example, herpesviruses) or integrate into the host genomic DNA (lower panel example, retroviruses and HBV).

Infectious agents can contribute to carcinogenesis by direct and/or indirect mechanisms (Figure 1). The direct-acting carcinogenic agents are generally found in a monoclonal form within the tumor cells. These agents help to keep the tumor phenotype through expression of either viral or cellular oncogenes (reviewed in [7]). Retroviruses, whose replication cycle requires the integration of the viral genome into the host genome, commonly transform because integration deregulates expression of cellular oncogenes or tumor suppressor genes (insertional mutagenesis, see Section 4.2). On the other hand, EBV is an example of a virus that does not need to integrate and transforms through expression of its own oncogenes.

**Figure 2:**
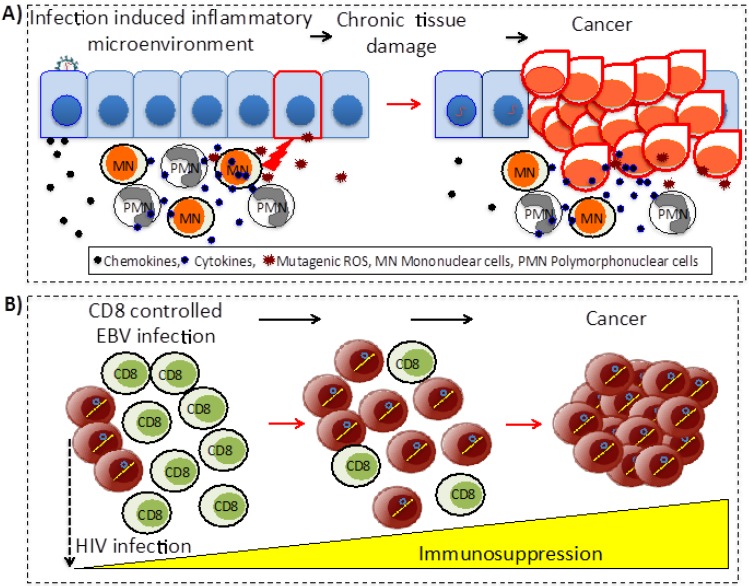
Indirect mechanisms of viral carcinogenesis. (**A**) Chronic inflammation. Infected cells produce chemokines attracting immune cells, which establish a chronic inflammatory microenvironment that persistently damage the local tissue. Cancer evolves within this cycle of infection, induced inflammation and tissue damage. (**B**) Immunosuppression. The prototype agent for immunosuppression is HIV. In immunocompetent individuals EBV infection is efficiently controlled by cytotoxic CD8 T cells; as HIV infection progresses and immune responses collapse, individuals become at increased risk of developing EBV associated lymphomas.

The indirect transforming viruses are not conditioned to exist within the cell that forms the tumor. These agents act through two main mechanisms: (i) triggering chronic inflammation and oxidative stress that persistently damage local tissues; and (ii) by producing immunosuppression that reduces or eliminates anti-tumor immune surveillance mechanisms (Figure 2). Among the most documented viral agents belonging to the first group are HBV and HCV; chronic inflammation produced by persistent infection associated with any of these viruses is a major risk to develop hepatocellular carcinoma (HCC) (reviewed in [15,16]). On the other hand, HIV belongs to the second group; patients with non-controlled infection and low T cell counts frequently develop lymphomas associated with EBV or KSV infection (reviewed in [17]).

Tumorigenic viruses were previously considered either exclusively direct or indirect transforming agents. However, some agents may require both mechanisms to induce carcinogenesis; for instance HBV and HCV [15,16]. *Helicobacter pylori* is the prototype indirect carcinogen through chronic inflammation [18]. Nevertheless, the bacterium also encodes the CagA oncoprotein, which is translocated to epithelial cells though a type IV secretion system (reviewed in [19]). Therefore, direct and indirect mechanisms are not mutually exclusive and some tissues may be equally dependent in both mechanisms for oncogenic transformation, such as the liver and stomach.

## 3. Human Oncogenic Viruses and Associated Cancers

Many different viruses have direct transformation characteristics; however, they have not been convincingly associated to human neoplasias based in the Bradford Hill criteria, and the IARC does not include them in the group of human type 1 carcinogens. Thus, adenoviruses, polyomaviruses SV40, JCV and BKV and others, will not be discussed here. The human cancers associated with viral infection are summarized in Table 2.

**Table 2:** Human oncogenic viruses and their associated tumors.

Human Virus	Associated Tumors	Reference
EBV	Burkitt’s lymphoma, Hodgkin’s lymphoma, immunosuppression-related lymphoma, T and NK cell lymphomas; nasopharyngeal and stomach carcinomas.	Reviewed in [11]
KSHV	Primary effusion lymphoma and Kaposi sarcoma	[20]
High-risk HPVs	Cervical, head and neck and anogenital tract carcinomas	Reviewed in [21]
MCPV	Merkel cell carcinoma	[22]
HBV	Hepatocellular carcinoma	[23]
HCV	Hepatocellular carcinoma	[24]
HTLV1	Adult T-cell leukemia/lymphoma	[25]

### 3.1. Herpesviruses: Epstein Barr Virus and Kaposi Sarcoma-Associated Herpesvirus

Herpesviruses are enveloped viruses with double-stranded linear DNA that after infecting the host cell remain in the nucleus as episomes (reviewed in [26]). Both EBV and KSHV show a biphasic life cycle consisting of a latent and a lytic phase. The latent phase seems to be the primary choice in which most of viral gene expression is shut down. This phase allows these viruses to coexist with the host generally asymptomatically and only in unusual situations may cause disease, e.g., during pharmacological or HIV induced immunosuppression. The lytic phase occurs in healthy individuals only in poorly understood sporadic events of reactivation.

EBV, also known as HHV4 (Human *Herpesvirus Type 4*), is found in approximately 95% of the adult population worldwide [27]; its principal routes of transmission are oral and blood [28,29], while intrauterine transmission has been documented too [30,31]. Early acquisition of this agent does not cause disease but when primary infection occurs during adolescence or early adulthood it causes infectious mononucleosis (reviewed in [32]). Interestingly, this condition represents a risk factor for developing Hodgkin’s lymphoma (reviewed in [33]).

B cells are the main target of EBV infection (reviewed in [34]); more rarely and less understood, EBV can also infect epithelial cells, mainly in the upper digestive tract, which is thought to occur in viral reactivation events [35]. EBV has mainly been associated with malignancies of B and epithelial cells of the upper digestive tract (Table 2), which provides biological plausibility and coherence to the role of EBV in these neoplasias.

EBV is found in a latent stage in both lymphomas and carcinomas, and within the latent genes, there are several with oncogenic properties. The viral protein best recognized as oncogenic is LMP1, a signaling protein that imitates a constitutively active TNF receptor. LMP1 activates MAP kinases and STAT and NFκB transcription factors in B cells, and also PI3K in epithelial cells [36]. LMP1 increases proliferation and survival of the infected cell. Of note, STAT and NFκB activation potentially stimulates expression of cytokines and chemokines important to establish the inflammatory microenvironment critical to create the niche from which infectious and non-infectious tumors emerge. LMP2A is another constitutively active viral protein with ITAM (*immunoreceptor tyrosine activated motif*) signaling domains [37]. LMP2A expression promotes the activation of PLCγ and PI3K pathways, which correlates with its capacity to transform epithelial cells and to confer a migratory phenotype to the transformed cell [38]. LMP1 and LMP2A provide antigen recognition-like signals to B cells, required for differentiation into long-lived memory cells in which the virus persists hidden from antagonistic immune responses. Although, both proteins can be expressed in EBV-induced carcinomas, their normal function in non-lymphoid tissue is not clear.

EBV-associated tumors are characterized by the expression of a different set of viral transcripts or latencies. In lymphomas arousing in immunosuppressed individuals (latency III) the family of EBNA proteins provides with additional oncogenic insults. For instance, EBNA-LP, -3A and -3C directly interfere with p53 and pRb functions, as well as with other proteins of the G0 to G1 phase transition. EBNA-1 is the common protein expressed in all EBV-associated neoplasias; it is expressed in latency III, latency II (Hodgkin’s lymphoma and carcinomas) and it is the sole viral protein expressed in latency I (Burkitt’s lymphoma). This absolute requirement for EBNA-1 is probably due to its capacity to equally segregate EBV episomes to both daughter cells during cell division [39].

The prevalence of KSHV infection varies among geographic regions, being 5% in Europe, Asia and some parts of North America and more than 50% in sub-Saharan Africa. KSHV is transmitted from casual contacts as well as through sexual contact, blood transfusion and organ transplant. In non‑endemic regions, the main via of transmission is probably through sexual contact and the use of contaminated syringes [40]. KSHV is the etiological agent of both lymphomas and sarcomas [41] (Table 2). Neoplasias associated with KSVH were not frequent before the AIDS pandemic, but currently represent one of the most important signs of this disease [42,43].

Several KSVH genes have potential oncogenic properties, for example, modulation of transduction of signals by K1 and K5; regulation of cell cycle by v-Cyclin and LANA 1; apoptosis inhibition by K1, vFLIP and v-Bcl2 and immune modulation by v-IRF, K3 and K5 (reviewed in [41]). LANA1 cooperates with h-Ras to transform fibroblasts and immortalize endothelial cells [44]. v-FLIP induces the development of lymphomas in transgenic mice primarily through its anti-apoptosis activity, which has been related to the activation of NF*k*B [45,46]. K1 also contains an ITAM signaling domain similar to the one found in EBV LMP2A and activates PI3K. K1 expressed in mice as a transgene promotes the development of sarcomas and lymphomas [47]. These similarities in the transformation mechanisms of both herpesviruses satisfy the principle of analogy of Bradford Hill’s causation criteria.

KSHV is often lytic in a small number of tumor cells [48], and some of its potentially oncogenic genes are products of the lytic cycle. Also similar to HCMV in gliomas, a few K1 lytic genes provide growth and angiogenic functions in a paracrine fashion, favoring tumor growth [49,50,51,52]. Of note, EBV defective viruses unable to switch to lytic cycle trigger less aggressive lymphomas in SCID mice than their wild type counterparts [53], and a small fraction of nasopharyngeal carcinomas (NPC) also harbors the virus in lytic phase [54]. Whether analogous mechanisms are in place for EBV, KSHV and HCMV in their paracrine requirement of lytic cycle proteins is not clear.

### 3.2. High-Risk Papillomaviruses

Human papilloma viruses belong to the *Papillomaviridae* family; they contain a double-strand DNA genome of approximately 8000 bp and are not enveloped viruses. More than 100 members of this family have been described and from them, more than a dozen (types 16, 18, 31, 33, 35, 45, 51, 52, 56, 58, 59, 62, 66 and 68) have been classified as high-risk due to their epidemiological association with cervical and other cancers (Table 2; reviewed in [21]). HPV subtypes 16 and 18 are the most frequently found in tumors; the first is mainly associated with invasive cervical cancer and the second is the most frequent in squamous cell carcinoma [55,56]. Low-risk HPVs generally cause benign lesions, such as warts (reviewed in [57]).

HPV is transmitted by skin contact, including genital contact during sexual intercourse; thus HPV infection in the genital area tents to be common in sexually active persons. Infection is generally controlled by the immune system and only in a low number of people, HPV persists, increasing the risk to develop epithelial lesions (reviewed in [58]). Viral persistence seems to be greatly helped by the inability of infected cells to present antigenic epitopes to adaptive immune cells, which is common in individuals with alterations in the HLA (Human Leucocyte Antigen) antigen presentation pathway (reviewed in [59]).

The neoplastic progression involves a series of histological changes that have been stratified in clinical stages, which correlate with differential expression of viral oncogenes and accumulation of mutations in the host genome. The main oncogenic proteins are E6 and E7, which are required since the first lesions and are necessary for the maintenance of the malignant phenotype. HPV is usually not integrated into the host genomic DNA, and E2 negatively regulates the expression of E6 and E7. An important event in the oncogenic process is the integration of the viral genome, a step usually resulting in loss of E2 and over-expression of E6 and E7 (reviewed in [60]). Increased expression of E6 and E7 correlates with progression to high grade lesions and eventually to carcinoma *in situ* (reviewed in [58]).

### 3.3. Merkel Cell Polyomavirus

Polyomaviruses are non-enveloped viruses with a circular, double-stranded DNA of approximately 5000 bp. The members of this family are present in all regions of the world infecting several species. Historically, it was considered that only JCV and BKV polyomaviruses infected humans, but next generation sequencing techniques have enabled the identification of at least nine other members in humans, among them MCPV. MCPV was identified in 2008 in an aggressive skin cancer denominated Merkel cell carcinoma (MCC) [22]. Virtually the whole adult population worldwide is infected by MCPV. Evidence supporting the participation of this agent in MCC carcinogenesis includes the presence of MCPV genomes in about 80% of the tumors but not in healthy tissue, and the clonal integration of the viral genome [22,61,62,63]. MCPV oncogenic transformation may result from loss of immune surveillance, as MCC mainly occurs in immunosuppressed individuals. MCC was a very rare cancer before the AIDS pandemia, and today, there are around 1700 new cases per year in the US [64,65].

The MCPV genome is inserted into the host genome during viral carcinogenesis. Integration is characterized by preserving the viral induced cell proliferation functions while abrogating viral replication; the latter probably due to deletion of some of the viral T antigen gene regions [66,67]. Viral integration also favors host resistance to cell death promoting viral persistence in a latent state [68]. This is a significant difference between the presence of the virus in MCC and in non-tumor tissue.

Due to the recent discovery of MCPV, we still do not understand the function of viral proteins. However, some viral proteins present homology in functional domains with tumorigenic polyomaviruses from non-human species. For example, like SV40 MCPV T antigens are generated by differential splicing to produce large T and small T antigens [69]. The large T antigen presents the structural motif that inactivates pRb (LXCXE) [70], and the T antigen is generally expressed in MCC, and even in its truncated form it maintains intact the pRb-inactivating domain [71]. Inactivation of the T antigen in MCC cell lines results in cell death, further supporting the causative role of MCPV in MCC [72]. Also, the small T antigen conserves the AKT/mTOR activating domain, which is responsible for loss of contact inhibition and promoting independent growth of substrate and serum [73].

### 3.4. Hepatitis B Virus

The *Hepadnaviridae* family groups a series of viruses that cause liver disease in animals, with Hepatitis B virus (HBV) infecting humans. HBV is an enveloped virus with an approximate 3.2 Kb genome of a partially double stranded DNA chain and a single stranded fragment. HBV replicates through an intermediary RNA via a viral reverse transcriptase. The main target of infection by HBV is the hepatocyte and infection can occur through vertical or horizontal transmission starting in the first years of life or during adulthood (reviewed in [74]).

Chronic infection by HBV is one of the main causes of hepatocellular carcinoma (HCC). The carcinogenesis process triggered by HBV is complex, involving direct and indirect mechanisms with the latter being driven by chronic inflammation (reviewed in [75]). Direct mechanisms such as expression of viral oncogenes and insertional mutagenesis have also been documented [76]. HBV X (HBx) is the main oncogenic viral protein. HBx is a viral replication protein that participates in transcription and DNA repair through which it regulates cell cycle, apoptosis and genomic instability [77]. Furthermore, HBx transgenic mice develop liver carcinomas [78].

### 3.5. Hepatitis C Virus

Hepatitis C virus (HCV) is a member of the *Flaviviridae* family; there are at least six genotypes that are regionally distributed and divided into subtypes [79]. The HCV genome consists of a single strand RNA of positive polarity of approximately 9600 nucleotides from which a polyprotein is translated from an unique open reading frame and later subdivided into different viral polypeptides by viral proteases (reviewed in [80]). HCV infects hepatocytes causing an acute infection that may turn chronic when the immune system cannot eliminate it. In those cases, the carrier may progress to hepatitis, cirrhosis and eventually to HCC (reviewed in [80]). It is estimated that more than 170 million persons worldwide are infected by HCV from which about 40% will develop some form of liver disease and 1%–4% HCC [81]. Transmission commonly occurs through blood and infected blood products.

Direct and indirect transforming mechanisms have also been described for HCV. The viral oncoprotein Core is the only viral product that in transgenic mice promotes the appearance of HCC [82]. Core is the main trigger of steatosis, an abnormal retention of lipids within the hepatocyte, and oxidative stress leading to chronic liver damage and HCC [83]. Different functions have been attributed to this protein, including altered cellular gene transcription, cell proliferation and cell death. For instance, Core expression correlates with changes in the activity of bona fide cellular tumor suppressors and oncogenes, and also of intermediaries of MAP kinases, NFκB and β-catenin signaling pathways [84]. Core protein regulates ROS production by inducing nitric oxide synthase (iNOS) which activates cyclooxygenase-2 (COX-2), importantly contributing with oxidative stress [85]. iNOS and COX-2 are also important components of the inflammatory pathway leading to cancer (reviewed in [86,87]). Core localizes in the mitochondria where it regulates levels of the mitochondrial chaperone prohibitin; it is proposed that altered binding of prohibitin and cytochrome c oxidase results in increased oxidative stress that favors DNA damage [88]. Taken together all these data has contributed to the formation of a model in which accelerated cell division by the inhibition of p53, pRb and other cell proteins in the presence of DNA damage by oxidative stress and the inflammatory response leads to the development of HCC.

### 3.6. Human T-Lymphotropic Virus Type 1

The *Retroviridae* family groups several viruses with two copies of a positive sense single stranded RNA genome that is retro-transcribed to DNA and integrated into the host cell genome. Retroviruses are classified as simple and complex. Simple retroviruses encode *gag*, *pol* and *env* genes from which structural proteins are expressed, plus other proteins involved in viral replication and integration. Complex viruses encode additional regulatory genes besides the mentioned above.

HTLV1 is a potent direct carcinogenic agent that has been associated with a spectrum of lymphoproliferative diseases collectively referred as adult T-cells leukemia/lymphoma (ATL) (reviewed in [89]). HTLV1 is endemic of Japan, the Western African coast, Central America and the Caribbean, with 15–25 million people infected worldwide [90]. There are three demonstrated ways of transmission for HTLV1: sexual contact, intravenous and breast feeding. The virus infects T- and B-lymphocytes and dendritic cells *in vivo*.

Although, the main retroviral mechanism of transformation is by insertional mutagenesis (see Section 4.2), HTLV1 is a complex retrovirus whose genome also encodes the Tax oncoprotein. Tax has the ability to immortalize cells *in vitro* and its enforced expression in transgenic mice results in development of leukemia/lymphoma [91,92,93,94,95]. Tax is a transcriptional activator/repressor capable of modulating expression of multiple cellular genes and it also directly interacts with a plethora of cellular proteins. Tax principal mechanism of transformation is related to reprogramming cell cycle and inhibition of DNA repair [96]. Tax induces NFκB activity, which stimulates the expression of cytokines and their receptors, including those of IL-13, IL-15, IL-2, IL-2Rα and co-stimulatory surface receptors (OX40/OX40L) [97,98,99]. Importantly, this activity mimics the chronic inflammatory process critical in the oncogenic progression of many types of cancers. These molecules trigger T cell proliferation, which may help to amplify the pool of HTLV1 infected cells. Thus, contrary to other cancers in which the inflammatory process is mediated by immune cells in response to the oncogenic insult, in HTLV1 infection this is directly induced by Tax. Besides NFkB promoters, Tax also regulates expression of cellular transcriptional promoters through interaction with cyclic-AMP response element binding protein (CREB) and serum response factor (SRF) (reviewed in [96]).

## 4. Common Mechanisms of Direct Carcinogenesis

### 4.1. Viral Oncogenes and Oncoproteins

#### 4.1.1. p53 and pRb Inactivation and Other Targets of Increased Proliferation and Survival

Viral oncogenes often increase the rate of cell proliferation and resistance to apoptosis, which eventually leads to alterations in DNA repair mechanisms and genomic instability. Increased mutation rates then alter cell polarity, with substrate-independent growth, and acquisition of cell migration properties, among other malignancy-associated features. The mechanisms used by viruses to induce these cellular changes are similar and often converge on common signaling pathways and transcription factors. For instance, inactivation of p53 and pRb tumor suppressor genes is an event that occurs in most pathways of viral oncogenesis, both human and animal (reviewed in [100,101,102]). In conditions of DNA damage, p53 arrests cell cycle until the damage has been repaired. When this does not occur, p53 induces cell apoptosis or cell senescence (reviewed in [103,104]). pRb also arrests cell cycle progression after binding and inactivating members of the E2F family of transcription factors [102]. pRb specifically inhibits the G1-S transition in response to DNA damage. Thus, an accumulation of mutations and chromosomal abnormalities is favored in the absence of p53 and pRb function. Since tumorigenic viruses are not usually associated with the massive production of viral infectious particles that characterize acute-infecting viruses, they relay in triggering cell proliferation mechanisms to increase the pool of infected cells. Furthermore, the termini of the viral genomes could be sensed as nicked DNA by the p53 and pRb machinery, and this would trigger host cell apoptosis immediately after infection, if both proteins were not inactivated.

HPV E6 and E7 induce the degradation of tumor suppressor proteins, p53 and pRb, respectively. E6 catalyzes the degradation of p53 by binding to the E6 associated protein (E6AP), a cellular protein with ubiquitin-ligase activity. The E6/E6AP complex binds to the p53 central region, which is then ubiquitinated and subsequently degraded in the proteasome [105,106]. E6 also blocks the cell cycle inhibitor p16^INK4^, which increases cell proliferation [107]. E7 directly induces release of E2F1-3 from pRb/E2F1-3 complexes, E2F1-3 in turn activates transcription of genes involved in cell cycle progression such as cyclins E and A [60,108]. HTLV1 Tax induces hyper-phosphorylation of pRb while promoting its degradation in the proteasome [109]. The mechanism by which Tax affects p53 function is less well understood and many mechanisms have been proposed, including hyper-phosphorylation, interfering with p53 function through competitive binding of cellular co-activators and through direct binding mediated by NFκB [110]. Tax also interferes with the activity and/or expression levels of cyclins and cyclin-dependent kinases [111,112,113]. Another HTLV1 protein, HBZ, induces over-expression of E2F1 target genes stimulating the proliferation of T lymphocytes [114]. KSHV LANA 1 inactivates p53 and it induces pRb phosphorylation and subsequent inactivation through its association with Cdk6 [115,116,117]. Most EBV latency III proteins target p53 and pRb for inactivation, along with other cell proliferation proteins: HA95, HAX1, cyclin A and D, p27kip1, p16INK4A and c-Myc [34].

Cell cycle progression and cell survival are conjointly regulated mechanisms. Still, tumor viruses often trigger additional survival mechanisms besides p53 and pRb inactivation. EBV LMP1 and LMP2A constitutively activate NFκB and PI3K/Akt signaling pathways, which results in increased activity of anti-apoptotic proteins Bcl-2, Bcl-xl, Mcl1 and A20 [118,119,120]. EBV also encodes BHRF1, a Bcl-2 homologue that in a subset of Burkitt’s lymphoma seems to counteract c-Myc pro-apoptotic activity [121]. HTLV1 Tax is also an important activator of NFκB and PI3K/Akt signaling pathways, and HTLV p12 and p13 proteins regulate Bcl-2 and caspase 3 and 9 activity [122]. HPV E6 and E7 function has been associated with degradation of pro-apoptotic proteins pro-caspase 8, FADD and BAK, and upregulation of expression of anti-apoptotic proteins c-IAP2 and survivin [123,124,125,126,127]. E6 binds to the E6-associated protein ligase (E6AP), an ubiquitin ligase that targets E6-interacting pro‑apoptotic proteins to the proteasome. HBV HBx interacts with Damaged DNA Binding Protein 1 (DDB1) inhibiting proteasome activity resulting in resistance to apoptosis [128]. Anti-apoptotic mechanisms have also been described for HCV core and NS5A proteins (reviewed in [129]). Because virally infected cells are at high risk of elimination by apoptosis, anti-apoptotic mechanisms are critical for viral persistence and carcinogenesis.

#### 4.1.2. Genomic Instability

Another common carcinogenic route promoted by infectious agents is genomic instability, which leads to gene amplification and deletion, changes in the number of chromosomes (polyploidy and aneuploidy) and aberrant fusion of non-homologous chromosomes (translocations). For instance, HPV-16 E6 and E7 proteins promote gene amplification, structural chromosomal alterations and centrosome replication errors leading to aneuploidy and polyploidy. Thus, HPV immortalized cell lines are characterized by gain and loss of whole chromosomes [130,131,132,133]. In agreement, aneuploidy can be found as early as in HPV-associated noninvasive lesions (reviewed in [134]). HBV HBx also interferes with genomic instability. HBx forms complexes with HBx interacting protein (HBXIP) altering the formation of the mitotic spindle and the centrosome function [77]. EBV EBNA-1 may promote genomic instability through activation of the recombinase-activating genes RAG1 and RAG2 [7], which may be responsible for the Myc chromosomal translocation present in Burkitt’s lymphoma [135]. Another enzyme associated with genomic instability is activation-induced cytidine deaminase (AID), whose expression is induced by EBV during the transit through germinal center reaction. Increased rate of mutations are observed in the variable regions of heavy and light chains after EBV infection [136]. Whether other host genomic regions are also targeted by AID is not know, but potentially this would facilitate EBV-induced transformation.

A mutator phenotype has also been attributed to Tax and both small and gross changes in DNA and chromosomes are often found in HTLV1 transformed cells [137,138]. On one hand, Tax multiple targets operating during the G2/M transition impair the DNA-damage-induced response, allowing cells to scape this transition with accumulated mutations [139]. On the other hand, Tax directly induces chromosomal instability by transcriptionally repressing various targets, including the DNA polymerase-β, an enzyme involved in base-excision repair [96]. Tax can also independently suppress the nucleotide excision repair mechanism, which is normally utilized by cells following UV irradiation [140]. Furthermore, ATL cells often contain an abnormal number of chromosomes (aneuploidy), and a role for Tax has been proposed, although the mechanism is not clear. Tax directly binds and inactivates MAD1, a mitotic spindle assembly checkpoint (SAC) kinetochore protein in charge of ensuring proper chromosomal segregation during mitosis [141]. Tax also promotes premature activation of the CDC20-associated anaphase promoting complex [142]. Overall these mechanisms would lead to faulty chromosomal segregation resulting in aneuploidy in HTLV1 infected cells.

#### 4.1.3. Interfering with Telomere Shortening

Telomere shortening and cell senescence are the natural consequence of unlimited cell proliferation, and tumor viruses also display mechanisms of telomere maintenance. Telomere length maintenance is a fine regulated mechanism involving a complex set of proteins and the enzyme telomerase (reviewed in [143]). Expression of telomerase in physiological conditions is restricted to cells with stem properties, e.g., germinal cells or somatic stem/progenitor cells, but telomerase expression is turned off in differentiated cells. How tumor viruses regulate telomere length is not clear, but HPV E6, EBV LMP1, KSHV LANA, HTLV1 Tax and HBV HBx have all been shown to induce expression of telomerase [144,145,146,147]. Tumor viruses interference with DNA repair mechanisms and concomitant genomic instability may be in great measure a consequence of bypassing regulatory checkpoints of telomere length and p53- and pRb-dependent senescence (reviewed in [148]). In this scenario, tumor viruses have evolved with these mechanisms in order to achieve replicative immortality and thus persistency.

#### 4.1.4. Interfering with Cell Polarity

Viral oncoproteins may also promote carcinogenesis by inactivating proteins related to cell polarity. Proteins containing PDZ (**p**ost synaptic density protein, drosophila **d**isc large tumor suppressor and **z**onula occludens-1 protein) domains function like scaffolds for both membrane and cytosolic supramolecular complexes, which have an important role in cell-cell contact and cell signaling. PDZ domains interact with target proteins through PBMs (PDZ domain-binding motif). A class I PBM was first described in the E4-ORF1 oncoprotein from adenovirus 9, and subsequently identified in other human virus oncoproteins, such as HPV E6 and HTLV1 Tax [149,150,151,152]. The E6 PBM is necessary for both *in vitro* and *in vivo* E6-mediated transformation [153,154]. On the other hand, mutational disruption of the Tax PBM reduces Tax-mediated cellular transformation and the capacity of HTLV1 to induce persistent infections [155,156]. Inactivation of cell polarity-associated proteins likely favors carcinogenesis by impairing morphogenesis, asymmetric division, migration and normal cell proliferation, survival and differentiation programs.

#### 4.1.5. Viral miRNAs

MicroRNAs (miRNAs) have recently being shown to also participate in cell transformation. miRNAs are strongly conserved single stranded RNAs of approximately 22 nucleotide long that regulate expression of most genes. miRNAs inhibit mRNA translation mainly by translational repression based on base pair complementarity (reviewed in [157,158]). Almost all cancers present altered expression of cellular miRNAs (reviewed in [159,160]). However, a new and interesting topic in viral oncology concerns to viruses encoding miRNAs with oncogenic capabilities. The first five viral miRNAs were described in the EBV positive B95 cell line; to date, more than 40 miRNAs produced from the EBV BARTs and BHFR1 transcripts have been identified [161,162]. Those miRNAs are able to inhibit apoptosis, and some target cellular tumor suppressor genes, such as: PUMA, Bin, TOMM22 and WIF1 [163,164,165,166]. EBV infection of gastric carcinoma cells (AGS) induced anchorage independence in absence of viral protein synthesis, highlighting the importance of EBV miRNAs in the malignant process [167].

### 4.2. Insertional Mutagenesis

In the retrovirus life cycle, the integrated viral genome (the provirus) is replicated as a cellular genetic element during the host cell cycle. Expression of the provirus is controlled by viral regulatory elements, the long terminal repeats (LTRs), which are powerful transcriptional activators that often control the expression of cellular genes in the vicinity of the insertion area. When the provirus is close to a cell proto-oncogene, the LTR can upregulate its expression to oncogenic results. Although, all best characterized examples of cell transformation are due to upregulation of proto-oncogenes, retroviruses can potentially also interrupt tumor suppressor genes with similar effects (reviewed in [168]). During viral progeny formation, infective particles sometimes carry cellular oncogenes that were close to the insertion site and which are transduced to new hosts, now under the control of the LTRs. Among those genes frequently transduced by retroviruses are cell receptors such as ErbB and Fms, kinases such as Src and Abl and transcription factors such as Jun, Fos and Myc. These chimerical viruses become non‑competent and unable to trigger lytic infections. Thus, these viruses have an augmented transforming capacity, as they are the only infectious agents capable of inducing tumors in just a few days. Due to this characteristic, such retroviruses are known as acute transformers. Preferential insertion sites are known as “hot spots”, for example, the murine mammary tumor virus (MMTV) responsible for breast cancer in several mouse species, frequently inserts in regions near to *Wnt* and *Notch* proto-oncogenes [169].

The mutagenesis mechanism by insertion or retro-transduction of cell proto-oncogenes has not been demonstrated in human retroviruses (HTLV1 mainly transforms by Tax expression. See Section 3.6) (reviewed in [170]). Next generation sequencing techniques have shown that HBV is preferably integrated in tumor cells in comparison to non-tumor infected hepatic tissue and its integration correlates with deregulated expression of *TERT*, *MLL4* and *CCNE* cellular oncogenes [76]. The similarity with animal retroviruses has granted analogy to this HBV mechanism of insertional mutagenesis. Although HPV and MCPV require integration to become oncogenic, in these cases the biological consequence is more similar to acute transforming retroviruses. Here, viral regulatory regions are lost and proviruses become defective and unable to produce infection competent progeny. Hence, integration correlates with the establishment of a latent stage, over-expression of viral oncogenes and host cell transformation (see Section 3.2 and Section 3.3).

## 5. Common Mechanisms of Indirect Carcinogenesis

**Figure 3:**
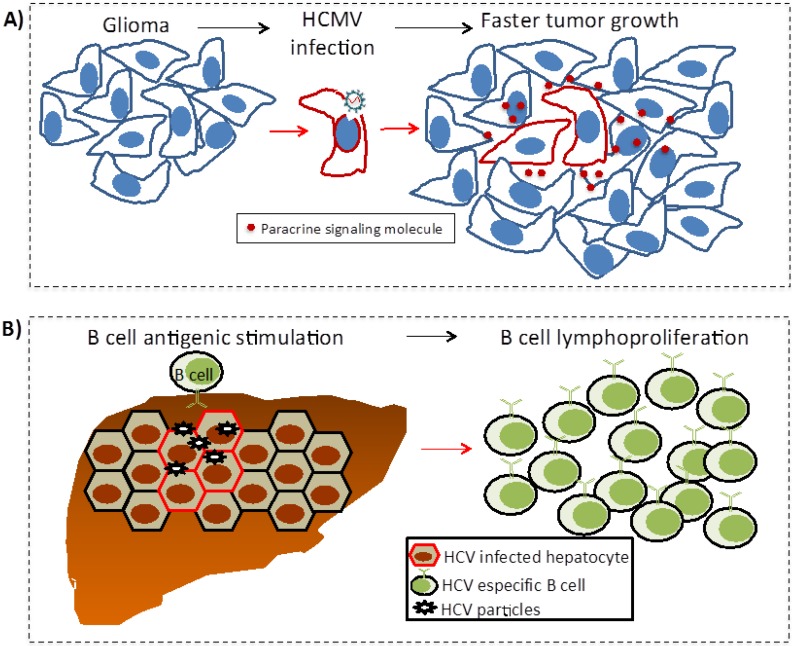
Other indirect mechanisms: Oncomodulation (**A**) and chronic antigen-driven lymphoproliferation (**B**). (**A**) In oncomodulation HCMV does not participate in the initial transformation of the glia; perhaps the virus has an increased tropism for tumor cells once the glioma has formed. Here, the virus only infects a fraction of the tumor cells activating signaling pathways that favor tumor growth; (**B**) B cells with antigen receptors specific for HCV antigens chronically respond to infected hepatocytes and free virus. This chronic stimulation increases the risk of unregulated lymphoproliferation and lymphoma.

The mechanisms of indirect oncogenesis are more difficult to demonstrate since they cannot be measured by *in vitro* assays, nor the expression of viral genes in transgenic animal models recapitulates the oncogenic process. These mechanisms have been proposed from epidemiologic evidence and coherence and plausibility principles are more difficult to fulfill. Besides chronic inflammation and immunosuppression (Figure 2), other proposed indirect mechanisms of transformation are described below (Figure 3).

### 5.1. Chronic Inflammation

Persistent infection is generally accompanied by local chronic inflammation, still in the presence of evasion mechanisms of the immune response. It has been proposed that this chronic inflammation induces a constant and progressive local damage, closely associated to regeneration events of the damaged tissue. The inflammatory response is characterized by local expression of pro-inflammatory cytokines, chemokines, adhesion molecules, growth factors and anti-apoptotic genes that regulate the sequential recruitment of leukocytes and stimulates fibroblasts and endothelial cells to divide and produce components of tissue remodeling and neovascularization (reviewed in [171]). A normal inflammatory response is self-limiting; chemoattraction of immune cells is gradually eliminated, pro-inflammatory cells already in the site of infection suffer apoptosis and are phagocytosed, while vascular changes are reversed. In contrast, in chronic inflammation associated with persistent infections, leukocytes remain in the lesion site and their apoptosis is suppressed. Additionally, to eliminate the infectious agent, immune cells produce oxygen and nitrogen free radicals, which are highly mutagenic. In this scenario, chronic inflammation favors the appearance of a cancerous clone, while tissue regeneration functions can also favor tumor growth, invasion and metastasis (for review, see [172], Figure 2A).

*Helicobacter pylori* is the prototype indirect carcinogen; it generates chronic gastric inflammation leading to gastric cancer through a series of progressive inflammatory lesions: non-atrophic gastritis, atrophic gastritis, intestinal metaplasia, dysplasia and gastric cancer [18]. *Helicobacter pylori* is also an IARC type I carcinogenic pathogen [173]. *Helicobacter pylori* stimulates the gastric epithelium to secrete IL-8 that attracts and activates neutrophils, favoring the establishment of a microenvironment populated by leukocytes and high concentrations of pro-inflammatory cytokines, such as TNF-α, IL-6, IL-1β and IL-12 [174]. Neutrophils release reactive oxygen species (superoxide anions, hydrogen peroxide, hydroxyl radicals and hydroperoxyl) and nitrogen oxides (nitric oxide, peroxynitrite and nitrogen dioxide) that induce mutations in cells of the gastric mucosa (reviewed in [175]). In agreement, individuals with IL-8, IL-1β and TNF-α polymorphisms present and increased risk to develop gastric cancer [176,177,178,179]. Of note, *Helicobacter pylori* pharmacological eradication in patients with pre-neoplastic lesions reverses tissue damage and halts the appearance of cancer, providing further support to the role of the bacterium in gastric cancer progression.

Although all oncogenic viruses maintain persistent infections, the role of inflammatory responses in oncogenesis is not clear. HCV and HBV triggered inflammation correlates with necrosis and tissue regeneration that eventually progresses to hepatic lesions such as steatosis, fibrosis and cirrhosis, from which liver cancer emerges. It has been observed that during steatosis and fibrosis, the liver is highly infiltrated by immune cells and there is a microenvironment of inflammatory cytokines and chemokines, among which TGF-β and IL-1β stand out (reviewed in [180]). We have also observed that increased EBV reactivation correlates with severe gastric inflammation and increased tissue damage leading to advanced gastric lesions, arguing for an important role for inflammation in the EBV‑associated transformation of the gastric mucosa [181,182].

### 5.2. Immunosuppression

The role of the immune system in onco-surveillance has been clearly established since the AIDS pandemic. Although, HIV is not capable of inducing tumors in its host cell, 40% of patients with AIDS develop cancers associated to the disease. Thus, severe immunosuppression induced by HIV infection indirectly promotes the development of tumors (reviewed in [17,183]). Individuals with a low CD8^+^ cytotoxic T lymphocyte count are more susceptible to infectious cancers [17], such as EBV- and KSHV-associated lymphomas (exemplified in Figure 2B), KSHV sarcomas, HPV head and neck and cervical carcinomas, and MCPV Merkel cell carcinomas. Due to these features, HIV is classified as an indirect carcinogenic agent, while, direct transformation mechanisms mediated by EBV, KSHV, HPV and MCPV are still operating.

A similar phenomenon is observed in individuals with pharmacological immunosuppression due to solid organ or bone marrow transplants. The post-transplant lymphoproliferative disorders (PTLD) are often EBV-associated B-cell proliferations. PTLDs begin as polyclonal proliferations with a high risk to evolve into monoclonal aggressive lymphomas [184,185]. Although, most PTLD arise from host lymphocytes, a donor origin is possible too. The use of T-cell depleting agents is a risk factor for EBV-positive PTLD, highlighting the importance of T-cells in the antitumor immunosurveillance mechanisms (reviewed in [186]). Infusion of autologous T-cells specific to the immunodominant EBV EBNA3A/3B/3C proteins has proven a very successful therapy [187,188].

### 5.3. Oncomodulation

There is evidence that viruses also participate in tumor growth modulating the biological course of an already-established cancer (Figure 3A). The term “oncomodulation” was suggested by Martin Michaelis *et al*. to describe the role of human cytomegalovirus (HCMV) in tumor progression [189]. HCMV is a herpesvirus whose worldwide prevalence is between 50% and 100% in the adult population; infection is normally asymptomatic and only produces disease under immunosuppressive conditions.

Up to today, there is not enough evidence of HCMV being capable of participating in the transformation process. Nevertheless, HCMV may infect tumor cells and through the expression of viral genes affecting signaling pathways important for proliferation, survival, angiogenesis, invasiveness and immune modulation, could increase the aggressiveness of the tumor [190,191,192,193,194]. The best documented example is HCMV participation in high degree gliomas, a brain cancer with extremely bad prognosis. HCMV genome and proteins have been found more frequently in high degree gliomas than in other central nervous system tumors or non-tumorous brain samples (e.g., from epilepsy) [195,196,197]. However, even when HCMV resides in the tumor, it only infects a fraction of the tumor cells and does not exhibit viral monoclonality; therefore, the HCMV association with high grade gliomas does not fulfill the Bradford Hill’s temporality criteria, arguing that infection happened after the event of transformation.

### 5.4. Chronic Antigen-Driven Lymphoproliferation

Cells of the immune system are expanded in response to infection; particularly, B cells exhibit extensive proliferation during the germinal center (GC) reaction in which they undergo antigen receptor isotype switch and increased target affinity (somatic hypermutation). The activation-induced cytidine deaminase (AID) is in charge of both processes in which the antigen receptor is modified. B cell lymphomas frequently emerge from the GC reaction due to the risky combination of increased proliferation and expression of mutagenic enzymes. EBV LMP1 and LMP2A provide decoy signals inducing infected B cells to go through the GC reaction and emerge as memory cells in which the virus can persist for the life-time of the host. EBV-associated lymphomas may partially result from this EBV-induced GC reaction [136]. Similarly, chronic antigenic stimulation resulting from other persistent infections can potentially increase the risk to develop lymphomas; among the most widely documented are, infection by bacteria *Helicobacter pylori*, *Borrelia burgdorferi*, *Campylobacter jejuni* and *Chlamydia psittaci* and by HCV. Concerning the latter, clonal B cell expansions have been observed in HCV infected patients correlating with longer chronic infections and with receptor specificity against HCV proteins [198,199,200]. Still, the most reliable evidence for a causal association comes from HCV pharmacological eradication, which is frequently associated with lymphoma remission [201]. Similarly, anti- *Helicobacter pylori* treatment results in regression of associated gastric MALT lymphomas [202].

## 6. Conclusions

The last 100 years have seen the birth and evolution of tumor virology with seven viral agents already been convincingly associated with the pathogenesis of cancer in humans. Tumor virology has importantly contributed to the understanding of the molecular mechanisms operating during carcinogenesis. However, causation is especially difficult to demonstrate because in most cases the tumor viruses are wide spread in the population without causing disease. It is essential to consider that infection by tumor viruses is never sufficient but always required for development of associated tumors. Cancer cannot be the aim of the virus since it compromises both host and virus survival. However, viral mechanisms of persistence in which cellular processes are impaired such as proliferation, survival, DNA repair, among others, provide a suitable substrate from which cancer can emerge after additional environmental aggressions and permissive host genetics. Today, Sir Austin Bradford Hill’s causation criteria are considered the experimental, epidemiological and clinical conceptual base from which to infer a virus-cancer causative relationship.

Two different modes of cellular transformation have been documented based in whether the virus is acting from within (directly) or outside (indirectly) the cell that will form the tumor. Direct mechanisms infer expression of viral oncogenes together with deregulation of cellular oncogenes and/or tumor suppressor genes. Among the most important indirect mechanisms are (i) the establishment of an inflammatory milieu in which chronic production of mutagenic molecules is persistently damaging the surrounding tissue, and (ii) immunosuppression with loss of the cancer immunosurveillance mechanisms. However, all tumor viruses probably present direct and indirect mechanisms and this separation mostly alludes to the main mechanism of cell transformation. The tumor microenvironment is always inflammatory whether infectious or aseptic, and inflammatory molecules importantly contribute with tumor initiation and progression. *Helicobacter pylori* is considered the prototype infectious agent transforming through chronic inflammation, still a bacterial oncogene able to induce gastric tumors in transgenic mice has recently been described. Similarly, EBV importantly cooperates with gastric inflammation and progression though a series of inflammatory lesions of increased severity [181,182] and HBV- and HCV-mediated liver cancer also progresses from a series of precursor inflammatory lesions besides their known capacity to express viral and cellular oncogenes. It is also possible that the contribution of direct and indirect mechanisms is mandated by the transforming tissue, with liver and gastric tissue equally depending on both modes of transformation.

Infection-associated tumors are responsible for 15%–20% of all cancer cases worldwide, representing an important challenge in public health programs. With the advent of new technologies it is highly probably that this frequency will increase. Wide genome sequencing technologies have recently allowed the discovery of MCPV and have helped to establish its causal association with Merkel cell carcinoma. Together with new tumor agents, it is probably that new mechanisms of infection-induced transformation will emerge while others could be better understood. The hit-and-run transforming mechanism proposes that a viral agent takes part in the carcinogenesis but it is later lost as the tumor cell acquires additional oncogenic hits [203]. This “non classical” oncogenic pathway is not compatible with current causality criteria. However, it is very likely that current causation criteria will be modified and extended in the future. For instance, Birdwell *et al*. used a model of transient infection in EBV-infected keratinocytes to analyze the pattern of methylation in CpG islands. They found that the epigenetic changes caused by infection correlated with a tumorigenic phenotype, which was maintained even after of loss of the virus [204]. Although, there is not evidence of transient infection by EBV does not happen naturally, the Birdwell’s work highlights the potential role of infections that are not maintained throughout cancer development. Also, facilitated by massive sequencing of tumor samples, a widespread APOBEC3B fingerprint was found among many types of cancers [205]. Because the APOBEC family of cytidine deaminases is part of an innate antiviral response, it is possible that this fingerprint reflects a history of past infections in which the antiviral response also collaterally triggered cellular somatic mutations leading to cancer.

The discovery of cancers with an infectious origin is critical to develop vaccines and preventive and therapeutic pharmacological therapies. This knowledge has already leaded to vaccines against HBV and high risk-HPV and targeted therapies against HIV and HCV.

## References

[1] Rous P. (1910). A transmissible avian neoplasm. (sarcoma of the common fowl.). J. Exp. Med..

[2] Rous P. (1911). A sarcoma of the fowl transmissible by an agent separable from the tumor cells. J. Exp. Med..

[3] Ellerman V., Bang O. (1908). Experimentelle leukämie bei hühnern. Zent. Bakteriol. Parasitenkd. Infectionskr. Hyg. Abt. Orig..

[4] Epstein M.A., Achong B.G., Barr Y.M. (1964). Virus particles in cultured lymphoblasts from burkitt’s lymphoma. Lancet.

[5] Durst M., Gissmann L., Ikenberg H., zur Hausen H. (1983). A papillomavirus DNA from a cervical carcinoma and its prevalence in cancer biopsy samples from different geographic regions. Proc. Natl. Acad. Sci. USA.

[6] Boshart M., Gissmann L., Ikenberg H., Kleinheinz A., Scheurlen W., zur Hausen H. (1984). A new type of papillomavirus DNA, its presence in genital cancer biopsies and in cell lines derived from cervical cancer. EMBO J..

[7] Moore P.S., Chang Y. (2010). Why do viruses cause cancer? Highlights of the first century of human tumour virology. Nat. Rev. Cancer.

[8] Parkin D.M. (2006). The global health burden of infection-associated cancers in the year 2002. Int. J. Cancer.

[9] Koch R. (1876). Untersuchungen über bakterien: V. Die ätiologie der milzbrand-krankheit, begründet auf die entwicklungsgeschichte des bacillus anthracis [investigations into bacteria: V. The etiology of anthrax, based on the ontogenesis of bacillus anthracis]. Cohns Beitr. Biol. Pflanz..

[10] Hill A.B. (1965). The environment and disease: Association or causation?. Proc. Royal Soc.Med..

[11] Thompson M.P., Kurzrock R. (2004). Epstein-barr virus and cancer. Clin. Cancer Res..

[12] Kulwichit W., Edwards R.H., Davenport E.M., Baskar J.F., Godfrey V., Raab-Traub N. (1998). Expression of the epstein-barr virus latent membrane protein 1 induces b cell lymphoma in transgenic mice. Proc. Natl. Acad. Sci. USA.

[13] Loeb L.A., Springgate C.F., Battula N. (1974). Errors in DNA replication as a basis of malignant changes. Cancer Res..

[14] Prindle M.J., Fox E.J., Loeb L.A. (2010). The mutator phenotype in cancer: Molecular mechanisms and targeting strategies. Curr. Drug Targets.

[15] Coleman W.B. (2003). Mechanisms of human hepatocarcinogenesis. Curr. Mol. Med..

[16] Zucman-Rossi J., Laurent-Puig P. (2007). Genetic diversity of hepatocellular carcinomas and its potential impact on targeted therapies. Pharmacogenomics.

[17] Chadburn A., Abdul-Nabi A.M., Teruya B.S., Lo A.A. (2013). Lymphoid proliferations associated with human immunodeficiency virus infection. Arch. Pathol. Lab. Med..

[18] Correa P., Piazuelo M.B. (2012). The gastric precancerous cascade. J. Dig. Dis..

[19] Hatakeyama M., Higashi H. (2005). *Helicobacter pylori* caga: A new paradigm for bacterial carcinogenesis. Cancer Sci..

[20] Chang Y., Cesarman E., Pessin M.S., Lee F., Culpepper J., Knowles D.M., Moore P.S. (1994). Identification of herpesvirus-like DNA sequences in aids-associated kaposi’s sarcoma. Science.

[21] Zur Hausen H. (2002). Papillomaviruses and cancer: From basic studies to clinical application. Nat. Rev. Cancer.

[22] Feng H., Shuda M., Chang Y., Moore P.S. (2008). Clonal integration of a polyomavirus in human merkel cell carcinoma. Science.

[23] Beasley R.P., Hwang L.Y., Lin C.C., Chien C.S. (1981). Hepatocellular carcinoma and hepatitis b virus. A prospective study of 22,707 men in taiwan. Lancet.

[24] Choo Q.L., Kuo G., Weiner A.J., Overby L.R., Bradley D.W., Houghton M. (1989). Isolation of a cDNA clone derived from a blood-borne non-a, non-b viral hepatitis genome. Science.

[25] Poiesz B.J., Ruscetti F.W., Gazdar A.F., Bunn P.A., Minna J.D., Gallo R.C. (1980). Detection and isolation of type c retrovirus particles from fresh and cultured lymphocytes of a patient with cutaneous t-cell lymphoma. Proc. Natl. Acad. Sci. USA.

[26] Rickinson A.B., Kieff E. (1996). Epstein-barr virus. Fields Virology.

[27] Henle G., Henle W., Clifford P., Diehl V., Kafuko G.W., Kirya B.G., Klein G., Morrow R.H., Munube G.M., Pike P. (1969). Antibodies to epstein-barr virus in burkittʼs lymphoma and control groups. J. Natl. Cancer Inst..

[28] Gerber P., Walsh J.H., Rosenblum E.N., Purcell R.H. (1969). Association of eb-virus infection with the post-perfusion syndrome. Lancet.

[29] Hoagland R.J. (1955). The transmission of infectious mononucleosis. Am. J. Med. Sci..

[30] Goldberg G.N., Fulginiti V.A., Ray C.G., Ferry P., Jones J.F., Cross H., Minnich L. (1981). In utero epstein-barr virus (infectious mononucleosis) infection. JAMA: J. Am. Med. Assoc..

[31] Meyohas M.C., Marechal V., Desire N., Bouillie J., Frottier J., Nicolas J.C. (1996). Study of mother-to-child epstein-barr virus transmission by means of nested pcrs. J. Virol..

[32] Stock I. (2013). Infectious mononucleosis—A “childhood disease” of great medical concern. Med. Mon. Pharma..

[33] Hjalgrim H. (2012). On the aetiology of hodgkin lymphoma. Dan. Med. J..

[34] Klein G., Klein E., Kashuba E. (2010). Interaction of epstein-barr virus (ebv) with human b-lymphocytes. Biochem. Biophys. Res. Commun..

[35] Morgan D.G., Niederman J.C., Miller G., Smith H.W., Dowaliby J.M. (1979). Site of epstein-barr virus replication in the oropharynx. Lancet.

[36] Shair K.H., Schnegg C.I., Raab-Traub N. (2008). Ebv latent membrane protein 1 effects on plakoglobin, cell growth, and migration. Cancer Res..

[37] Caldwell R.G., Wilson J.B., Anderson S.J., Longnecker R. (1998). Epstein-barr virus lmp2a drives b cell development and survival in the absence of normal b cell receptor signals. Immunity.

[38] Scholle F., Bendt K.M., Raab-Traub N. (2000). Epstein-barr virus lmp2a transforms epithelial cells, inhibits cell differentiation, and activates akt. J. Virol..

[39] Yates J.L., Warren N., Sugden B. (1985). Stable replication of plasmids derived from epstein-barr virus in various mammalian cells. Nature.

[40] Dukers N.H., Rezza G. (2003). Human herpesvirus 8 epidemiology: What we do and do not know. Aids.

[41] Cai Q., Verma S.C., Lu J., Robertson E.S. (2010). Molecular biology of kaposi’s sarcoma-associated herpesvirus and related oncogenesis. Adv. Virus Res..

[42] Viejo-Borbolla A., Ottinger M., Schulz T.F. (2004). Human herpesvirus 8: Biology and role in the pathogenesis of kaposi’s sarcoma and other aids-related malignancies. Curr. HIV/AIDS Rep..

[43] Beral V., Peterman T.A., Berkelman R.L., Jaffe H.W. (1990). Kaposi’s sarcoma among persons with aids: A sexually transmitted infection?. Lancet.

[44] Radkov S.A., Kellam P., Boshoff C. (2000). The latent nuclear antigen of kaposi sarcoma-associated herpesvirus targets the retinoblastoma-e2f pathway and with the oncogene hras transforms primary rat cells. Nat. Med..

[45] Chugh P., Matta H., Schamus S., Zachariah S., Kumar A., Richardson J.A., Smith A.L., Chaudhary P.M. (2005). Constitutive nf-kappab activation, normal fas-induced apoptosis, and increased incidence of lymphoma in human herpes virus 8 k13 transgenic mice. Proc. Natl. Acad. Sci. USA.

[46] Sun Q., Zachariah S., Chaudhary P.M. (2003). The human herpes virus 8-encoded viral flice-inhibitory protein induces cellular transformation via nf-kappab activation. J. Biol. Chem..

[47] Wang L., Dittmer D.P., Tomlinson C.C., Fakhari F.D., Damania B. (2006). Immortalization of primary endothelial cells by the k1 protein of kaposi’s sarcoma-associated herpesvirus. Cancer Res..

[48] Staskus K.A., Zhong W., Gebhard K., Herndier B., Wang H., Renne R., Beneke J., Pudney J., Anderson D.J., Ganem D. (1997). Kaposi’s sarcoma-associated herpesvirus gene expression in endothelial (spindle) tumor cells. J. Virol..

[49] Xie J., Pan H., Yoo S., Gao S.J. (2005). Kaposi’s sarcoma-associated herpesvirus induction of ap-1 and interleukin 6 during primary infection mediated by multiple mitogen-activated protein kinase pathways. J. Virol..

[50] Naranatt P.P., Krishnan H.H., Svojanovsky S.R., Bloomer C., Mathur S., Chandran B. (2004). Host gene induction and transcriptional reprogramming in kaposi’s sarcoma-associated herpesvirus (kshv/hhv-8)-infected endothelial, fibroblast, and b cells: Insights into modulation events early during infection. Cancer Res..

[51] Masood R., Cesarman E., Smith D.L., Gill P.S., Flore O. (2002). Human herpesvirus-8-transformed endothelial cells have functionally activated vascular endothelial growth factor/vascular endothelial growth factor receptor. Am. J. Pathol..

[52] Cerimele F., Curreli F., Ely S., Friedman-Kien A.E., Cesarman E., Flore O. (2001). Kaposi’s sarcoma-associated herpesvirus can productively infect primary human keratinocytes and alter their growth properties. J. Virol..

[53] Hong G.K., Gulley M.L., Feng W.H., Delecluse H.J., Holley-Guthrie E., Kenney S.C. (2005). Epstein-barr virus lytic infection contributes to lymphoproliferative disease in a scid mouse model. J. Virol..

[54] Martel-Renoir D., Grunewald V., Touitou R., Schwaab G., Joab I. (1995). Qualitative analysis of the expression of epstein-barr virus lytic genes in nasopharyngeal carcinoma biopsies. J. Gen. Virol..

[55] Munoz N., Bosch F.X., de Sanjose S., Herrero R., Castellsague X., Shah K.V., Snijders P.J., Meijer C.J. (2003). International Agency for Research on Cancer Multicenter Cervical Cancer Study, G. Epidemiologic classification of human papillomavirus types associated with cervical cancer. N. Engl. J. Med..

[56] Clifford G.M., Smith J.S., Plummer M., Munoz N., Franceschi S. (2003). Human papillomavirus types in invasive cervical cancer worldwide: A meta-analysis. Br. J. Cancer.

[57] Chow L.T., Broker T.R. (2013). Human papillomavirus infections: Warts or cancer?. Cold Spring Harbor Perspect. Biol..

[58] Woodman C.B., Collins S.I., Young L.S. (2007). The natural history of cervical hpv infection: Unresolved issues. Nat. Rev. Cancer.

[59] Zur Hausen H. (2008). Papillomaviruses—To vaccination and beyond. Biochem. Biokhimiia.

[60] Ghittoni R., Accardi R., Hasan U., Gheit T., Sylla B., Tommasino M. (2010). The biological properties of e6 and e7 oncoproteins from human papillomaviruses. Virus Genes.

[61] Becker J.C., Houben R., Ugurel S., Trefzer U., Pfohler C., Schrama D. (2009). Mc polyomavirus is frequently present in merkel cell carcinoma of european patients. J. Investig. Dermatol..

[62] Carter J.J., Paulson K.G., Wipf G.C., Miranda D., Madeleine M.M., Johnson L.G., Lemos B.D., Lee S., Warcola A.H., Iyer J.G. (2009). Association of merkel cell polyomavirus-specific antibodies with merkel cell carcinoma. J. Natl. Cancer Inst..

[63] Duncavage E.J., Zehnbauer B.A., Pfeifer J.D. (2009). Prevalence of merkel cell polyomavirus in merkel cell carcinoma. Mod. Pathol..

[64] Agelli M., Clegg L.X. (2003). Epidemiology of primary merkel cell carcinoma in the United States. J. Am. Acad. Dermatol..

[65] Hodgson N.C. (2005). Merkel cell carcinoma: Changing incidence trends. J. Surg. Oncol..

[66] Kassem A., Schopflin A., Diaz C., Weyers W., Stickeler E., Werner M., Zur Hausen A. (2008). Frequent detection of merkel cell polyomavirus in human merkel cell carcinomas and identification of a unique deletion in the vp1 gene. Cancer Res..

[67] Kwun H.J., Guastafierro A., Shuda M., Meinke G., Bohm A., Moore P.S., Chang Y. (2009). The minimum replication origin of merkel cell polyomavirus has a unique large t-antigen loading architecture and requires small t-antigen expression for optimal replication. J. Virol..

[68] Moens U., van Ghelue M., Johannessen M. (2007). Oncogenic potentials of the human polyomavirus regulatory proteins. Cell. Mol. Life Sci..

[69] Shuda M., Arora R., Kwun H.J., Feng H., Sarid R., Fernandez-Figueras M.T., Tolstov Y., Gjoerup O., Mansukhani M.M., Swerdlow S.H. (2009). Human merkel cell polyomavirus infection i. Mcv t antigen expression in merkel cell carcinoma, lymphoid tissues and lymphoid tumors. Int. J. Cancer.

[70] Shuda M., Feng H., Kwun H.J., Rosen S.T., Gjoerup O., Moore P.S., Chang Y. (2008). T antigen mutations are a human tumor-specific signature for merkel cell polyomavirus. Proc. Natl. Acad. Sci. USA.

[71] Houben R., Adam C., Baeurle A., Hesbacher S., Grimm J., Angermeyer S., Henzel K., Hauser S., Elling R., Brocker E.B. (2012). An intact retinoblastoma protein-binding site in merkel cell polyomavirus large t antigen is required for promoting growth of merkel cell carcinoma cells. Int. J. Cancer.

[72] Houben R., Shuda M., Weinkam R., Schrama D., Feng H., Chang Y., Moore P.S., Becker J.C. (2010). Merkel cell polyomavirus-infected merkel cell carcinoma cells require expression of viral t antigens. J. Virol..

[73] Shuda M., Kwun H.J., Feng H., Chang Y., Moore P.S. (2011). Human merkel cell polyomavirus small t antigen is an oncoprotein targeting the 4e-bp1 translation regulator. J. Clin. Investig..

[74] Kew M.C. (2010). Epidemiology of chronic hepatitis b virus infection, hepatocellular carcinoma, and hepatitis b virus-induced hepatocellular carcinoma. Pathol.-Biol..

[75] Tarocchi M., Polvani S., Marroncini G., Galli A. (2014). Molecular mechanism of hepatitis b virus-induced hepatocarcinogenesis. World J. Gastroenterol..

[76] Sung W.K., Zheng H., Li S., Chen R., Liu X., Li Y., Lee N.P., Lee W.H., Ariyaratne P.N., Tennakoon C. (2012). Genome-wide survey of recurrent hbv integration in hepatocellular carcinoma. Nat. Genet..

[77] Wen Y., Golubkov V.S., Strongin A.Y., Jiang W., Reed J.C. (2008). Interaction of hepatitis b viral oncoprotein with cellular target hbxip dysregulates centrosome dynamics and mitotic spindle formation. J. Biol. Chem..

[78] Kim C.M., Koike K., Saito I., Miyamura T., Jay G. (1991). Hbx gene of hepatitis b virus induces liver cancer in transgenic mice. Nature.

[79] Simmonds P., Bukh J., Combet C., Deleage G., Enomoto N., Feinstone S., Halfon P., Inchauspe G., Kuiken C., Maertens G. (2005). Consensus proposals for a unified system of nomenclature of hepatitis c virus genotypes. Hepatology.

[80] Tang H., Grise H. (2009). Cellular and molecular biology of hcv infection and hepatitis. Clin. Sci..

[81] Morgan R.L., Baack B., Smith B.D., Yartel A., Pitasi M., Falck-Ytter Y. (2013). Eradication of hepatitis c virus infection and the development of hepatocellular carcinoma: A meta-analysis of observational studies. Ann. Intern. Med..

[82] Moriya K., Fujie H., Shintani Y., Yotsuyanagi H., Tsutsumi T., Ishibashi K., Matsuura Y., Kimura S., Miyamura T., Koike K. (1998). The core protein of hepatitis c virus induces hepatocellular carcinoma in transgenic mice. Nat. Med..

[83] Moriya K., Yotsuyanagi H., Shintani Y., Fujie H., Ishibashi K., Matsuura Y., Miyamura T., Koike K. (1997). Hepatitis c virus core protein induces hepatic steatosis in transgenic mice. J. Gen. Virol..

[84] Pei Y., Zhang T., Renault V., Zhang X. (2009). An overview of hepatocellular carcinoma study by omics-based methods. Acta Biochim. Biophys. Sin..

[85] Nunez O., Fernandez-Martinez A., Majano P.L., Apolinario A., Gomez-Gonzalo M., Benedicto I., Lopez-Cabrera M., Bosca L., Clemente G., Garcia-Monzon C. (2004). Increased intrahepatic cyclooxygenase 2, matrix metalloproteinase 2, and matrix metalloproteinase 9 expression is associated with progressive liver disease in chronic hepatitis c virus infection: Role of viral core and ns5a proteins. Gut.

[86] Murakami A., Ohigashi H. (2007). Targeting nox, inos and cox-2 in inflammatory cells: Chemoprevention using food phytochemicals. Int. J. Cancer.

[87] Ohshima H., Tazawa H., Sylla B.S., Sawa T. (2005). Prevention of human cancer by modulation of chronic inflammatory processes. Mutat. Res..

[88] Fujinaga H., Tsutsumi T., Yotsuyanagi H., Moriya K., Koike K. (2011). Hepatocarcinogenesis in hepatitis c: Hcv shrewdly exacerbates oxidative stress by modulating both production and scavenging of reactive oxygen species. Oncology.

[89] Kannian P., Green P.L. (2010). Human t lymphotropic virus type 1 (htlv-1): Molecular biology and oncogenesis. Viruses.

[90] Proietti F.A., Carneiro-Proietti A.B., Catalan-Soares B.C., Murphy E.L. (2005). Global epidemiology of htlv-i infection and associated diseases. Oncogene.

[91] Grassmann R., Dengler C., Muller-Fleckenstein I., Fleckenstein B., McGuire K., Dokhelar M.C., Sodroski J.G., Haseltine W.A. (1989). Transformation to continuous growth of primary human t lymphocytes by human t-cell leukemia virus type i x-region genes transduced by a herpesvirus saimiri vector. Proc. Natl. Acad. Sci. USA.

[92] Grassmann R., Berchtold S., Radant I., Alt M., Fleckenstein B., Sodroski J.G., Haseltine W.A., Ramstedt U. (1992). Role of human t-cell leukemia virus type 1 x region proteins in immortalization of primary human lymphocytes in culture. J. Virol..

[93] Akagi T., Shimotohno K. (1993). Proliferative response of tax1-transduced primary human t cells to anti-cd3 antibody stimulation by an interleukin-2-independent pathway. J. Virol..

[94] Hasegawa H., Sawa H., Lewis M.J., Orba Y., Sheehy N., Yamamoto Y., Ichinohe T., Tsunetsugu-Yokota Y., Katano H., Takahashi H. (2006). Thymus-derived leukemia-lymphoma in mice transgenic for the tax gene of human t-lymphotropic virus type i. Nat. Med..

[95] Benvenisty N., Ornitz D.M., Bennett G.L., Sahagan B.G., Kuo A., Cardiff R.D., Leder P. (1992). Brain tumours and lymphomas in transgenic mice that carry htlv-i ltr/c-myc and ig/tax genes. Oncogene.

[96] Azran I., Schavinsky-Khrapunsky Y., Aboud M. (2004). Role of tax protein in human t-cell leukemia virus type-i leukemogenicity. Retrovirology.

[97] Li X.H., Gaynor R.B. (2000). Mechanisms of nf-kappab activation by the htlv type 1 tax protein. AIDS Res. Hum. Retrovir..

[98] Harhaj E.W., Harhaj N.S., Grant C., Mostoller K., Alefantis T., Sun S.C., Wigdahl B. (2005). Human t cell leukemia virus type i tax activates cd40 gene expression via the nf-kappa b pathway. Virology.

[99] Mori N., Fujii M., Cheng G., Ikeda S., Yamasaki Y., Yamada Y., Tomonaga M., Yamamoto N. (2001). Human t-cell leukemia virus type i tax protein induces the expression of anti-apoptotic gene bcl-xl in human t-cells through nuclear factor-kappab and c-amp responsive element binding protein pathways. Virus Genes.

[100] Hollstein M., Sidransky D., Vogelstein B., Harris C.C. (1991). P53 mutations in human cancers. Science.

[101] Levine A.J., Momand J., Finlay C.A. (1991). The p53 tumour suppressor gene. Nature.

[102] Khidr L., Chen P.L. (2006). Rb, the conductor that orchestrates life, death and differentiation. Oncogene.

[103] Lee J.M., Bernstein A. (1995). Apoptosis, cancer and the p53 tumour suppressor gene. Cancer Metastasis Rev..

[104] Amundson S.A., Myers T.G., Fornace A.J. (1998). Roles for p53 in growth arrest and apoptosis: Putting on the brakes after genotoxic stress. Oncogene.

[105] Scheffner M., Werness B.A., Huibregtse J.M., Levine A.J., Howley P.M. (1990). The e6 oncoprotein encoded by human papillomavirus types 16 and 18 promotes the degradation of p53. Cell.

[106] Scheffner M., Huibregtse J.M., Vierstra R.D., Howley P.M. (1993). The hpv-16 e6 and e6-ap complex functions as a ubiquitin-protein ligase in the ubiquitination of p53. Cell.

[107] Reznikoff C.A., Yeager T.R., Belair C.D., Savelieva E., Puthenveettil J.A., Stadler W.M. (1996). Elevated p16 at senescence and loss of p16 at immortalization in human papillomavirus 16 e6, but not e7, transformed human uroepithelial cells. Cancer Res..

[108] Dyson N., Howley P.M., Munger K., Harlow E. (1989). The human papilloma virus-16 e7 oncoprotein is able to bind to the retinoblastoma gene product. Science.

[109] Giam C.Z., Jeang K.T. (2007). Htlv-1 tax and adult t-cell leukemia. Front. Biosci.: J. Virtual Libr..

[110] Pise-Masison C.A., Brady J.N. (2005). Setting the stage for transformation: Htlv-1 tax inhibition of p53 function. Front. Biosci..

[111] Haller K., Wu Y., Derow E., Schmitt I., Jeang K.T., Grassmann R. (2002). Physical interaction of human t-cell leukemia virus type 1 tax with cyclin-dependent kinase 4 stimulates the phosphorylation of retinoblastoma protein. Mol. Cell. Biol..

[112] Suzuki T., Narita T., Uchida-Toita M., Yoshida M. (1999). Down-regulation of the ink4 family of cyclin-dependent kinase inhibitors by tax protein of htlv-1 through two distinct mechanisms. Virology.

[113] Suzuki T., Kitao S., Matsushime H., Yoshida M. (1996). Htlv-1 tax protein interacts with cyclin-dependent kinase inhibitor p16ink4a and counteracts its inhibitory activity towards cdk4. EMBO J..

[114] Satou Y., Yasunaga J., Yoshida M., Matsuoka M. (2006). Htlv-i basic leucine zipper factor gene mrna supports proliferation of adult t cell leukemia cells. Proc. Natl. Acad. Sci. USA.

[115] Cai Q.L., Knight J.S., Verma S.C., Zald P., Robertson E.S. (2006). Ec5s ubiquitin complex is recruited by kshv latent antigen lana for degradation of the vhl and p53 tumor suppressors. PLoS Pathog..

[116] Friborg J., Kong W., Hottiger M.O., Nabel G.J. (1999). P53 inhibition by the lana protein of kshv protects against cell death. Nature.

[117] Si H., Robertson E.S. (2006). Kaposi’s sarcoma-associated herpesvirus-encoded latency-associated nuclear antigen induces chromosomal instability through inhibition of p53 function. J. Virol..

[118] Spender L.C., Cannell E.J., Hollyoake M., Wensing B., Gawn J.M., Brimmell M., Packham G., Farrell P.J. (1999). Control of cell cycle entry and apoptosis in b lymphocytes infected by epstein-barr virus. J. Virol..

[119] Mei Y.P., Zhou J.M., Wang Y., Huang H., Deng R., Feng G.K., Zeng Y.X., Zhu X.F. (2007). Silencing of lmp1 induces cell cycle arrest and enhances chemosensitivity through inhibition of akt signaling pathway in ebv-positive nasopharyngeal carcinoma cells. Cell Cycle.

[120] Portis T., Longnecker R. (2004). Epstein-barr virus (ebv) lmp2a mediates b-lymphocyte survival through constitutive activation of the ras/pi3k/akt pathway. Oncogene.

[121] Desbien A.L., Kappler J.W., Marrack P. (2009). The epstein-barr virus bcl-2 homolog, bhrf1, blocks apoptosis by binding to a limited amount of bim. Proc. Natl. Acad. Sci. USA.

[122] Saggioro D., Silic-Benussi M., Biasiotto R., D’Agostino D.M., Ciminale V. (2009). Control of cell death pathways by htlv-1 proteins. Front. Biosci..

[123] Underbrink M.P., Howie H.L., Bedard K.M., Koop J.I., Galloway D.A. (2008). E6 proteins from multiple human betapapillomavirus types degrade bak and protect keratinocytes from apoptosis after uvb irradiation. J. Virol..

[124] Garnett T.O., Filippova M., Duerksen-Hughes P.J. (2006). Accelerated degradation of fadd and procaspase 8 in cells expressing human papilloma virus 16 e6 impairs trail-mediated apoptosis. Cell Death Differ..

[125] Yuan H., Fu F., Zhuo J., Wang W., Nishitani J., An D.S., Chen I.S., Liu X. (2005). Human papillomavirus type 16 e6 and e7 oncoproteins upregulate c-iap2 gene expression and confer resistance to apoptosis. Oncogene.

[126] Filippova M., Parkhurst L., Duerksen-Hughes P.J. (2004). The human papillomavirus 16 e6 protein binds to fas-associated death domain and protects cells from fas-triggered apoptosis. J. Biol. Chem..

[127] Du J., Chen G.G., Vlantis A.C., Chan P.K., Tsang R.K., van Hasselt C.A. (2004). Resistance to apoptosis of hpv 16-infected laryngeal cancer cells is associated with decreased bak and increased bcl-2 expression. Cancer Lett..

[128] Becker S.A., Lee T.H., Butel J.S., Slagle B.L. (1998). Hepatitis b virus x protein interferes with cellular DNA repair. J. Virol..

[129] Aweya J.J., Tan Y.J. (2011). Modulation of programmed cell death pathways by the hepatitis c virus. Front. Biosci..

[130] Plug-DeMaggio A.W., Sundsvold T., Wurscher M.A., Koop J.I., Klingelhutz A.J., McDougall J.K. (2004). Telomere erosion and chromosomal instability in cells expressing the hpv oncogene 16e6. Oncogene.

[131] Duensing S., Munger K. (2002). The human papillomavirus type 16 e6 and e7 oncoproteins independently induce numerical and structural chromosome instability. Cancer Res..

[132] Duensing S., Duensing A., Crum C.P., Munger K. (2001). Human papillomavirus type 16 e7 oncoprotein-induced abnormal centrosome synthesis is an early event in the evolving malignant phenotype. Cancer Res..

[133] Duensing S., Lee L.Y., Duensing A., Basile J., Piboonniyom S., Gonzalez S., Crum C.P., Munger K. (2000). The human papillomavirus type 16 e6 and e7 oncoproteins cooperate to induce mitotic defects and genomic instability by uncoupling centrosome duplication from the cell division cycle. Proc. Natl. Acad. Sci. USA.

[134] Chen J.J. (2010). Genomic instability induced by human papillomavirus oncogenes. North Am. J. Med. Sci..

[135] Bornkamm G.W. (2009). Epstein-barr virus and its role in the pathogenesis of burkitt’s lymphoma: An unresolved issue. Semin. Cancer Biol..

[136] Heath E., Begue-Pastor N., Chaganti S., Croom-Carter D., Shannon-Lowe C., Kube D., Feederle R., Delecluse H.J., Rickinson A.B., Bell A.I. (2012). Epstein-barr virus infection of naive b cells *in vitro* frequently selects clones with mutated immunoglobulin genotypes: Implications for virus biology. PLoS Pathog..

[137] Marriott S.J., Lemoine F.J., Jeang K.T. (2002). Damaged DNA and miscounted chromosomes: Human t cell leukemia virus type i tax oncoprotein and genetic lesions in transformed cells. J. Biomed. Sci..

[138] Lemoine F.J., Marriott S.J. (2002). Genomic instability driven by the human t-cell leukemia virus type i (htlv-i) oncoprotein, tax. Oncogene.

[139] Chandhasin C., Ducu R.I., Berkovich E., Kastan M.B., Marriott S.J. (2008). Human t-cell leukemia virus type 1 tax attenuates the atm-mediated cellular DNA damage response. J. Virol..

[140] Kao S.Y., Lemoine F.J., Marriott S.J. (2001). P53-independent induction of apoptosis by the htlv-i tax protein following uv irradiation. Virology.

[141] Jin D.Y., Spencer F., Jeang K.T. (1998). Human t cell leukemia virus type 1 oncoprotein tax targets the human mitotic checkpoint protein mad1. Cell.

[142] Liu B., Hong S., Tang Z., Yu H., Giam C.Z. (2005). Htlv-i tax directly binds the cdc20-associated anaphase-promoting complex and activates it ahead of schedule. Proc. Natl. Acad. Sci. USA.

[143] Cohen S.B., Graham M.E., Lovrecz G.O., Bache N., Robinson P.J., Reddel R.R. (2007). Protein composition of catalytically active human telomerase from immortal cells. Science.

[144] Terrin L., dal Col J., Rampazzo E., Zancai P., Pedrotti M., Ammirabile G., Bergamin S., Rizzo S., Dolcetti R., de Rossi A. (2008). Latent membrane protein 1 of epstein-barr virus activates the htert promoter and enhances telomerase activity in b lymphocytes. J. Virol..

[145] Zhang X., Dong N., Zhang H., You J., Wang H., Ye L. (2005). Effects of hepatitis b virus x protein on human telomerase reverse transcriptase expression and activity in hepatoma cells. J. Lab. Clin. Med..

[146] Verma S.C., Borah S., Robertson E.S. (2004). Latency-associated nuclear antigen of kaposi’s sarcoma-associated herpesvirus up-regulates transcription of human telomerase reverse transcriptase promoter through interaction with transcription factor sp1. J. Virol..

[147] Gewin L., Myers H., Kiyono T., Galloway D.A. (2004). Identification of a novel telomerase repressor that interacts with the human papillomavirus type-16 e6/e6-ap complex. Genes Dev..

[148] Chen X., Kamranvar S.A., Masucci M.G. (2014). Tumor viruses and replicative immortality—Avoiding the telomere hurdle. Semin. Cancer Biol..

[149] Ohashi M., Sakurai M., Higuchi M., Mori N., Fukushi M., Oie M., Coffey R.J., Yoshiura K., Tanaka Y., Uchiyama M. (2004). Human t-cell leukemia virus type 1 tax oncoprotein induces and interacts with a multi-pdz domain protein, magi-3. Virology.

[150] Glaunsinger B.A., Lee S.S., Thomas M., Banks L., Javier R. (2000). Interactions of the pdz-protein magi-1 with adenovirus e4-orf1 and high-risk papillomavirus e6 oncoproteins. Oncogene.

[151] Rousset R., Fabre S., Desbois C., Bantignies F., Jalinot P. (1998). The c-terminus of the htlv-1 tax oncoprotein mediates interaction with the pdz domain of cellular proteins. Oncogene.

[152] Lee S.S., Weiss R.S., Javier R.T. (1997). Binding of human virus oncoproteins to hdlg/sap97, a mammalian homolog of the drosophila discs large tumor suppressor protein. Proc. Natl. Acad. Sci. USA.

[153] Spanos W.C., Hoover A., Harris G.F., Wu S., Strand G.L., Anderson M.E., Klingelhutz A.J., Hendriks W., Bossler A.D., Lee J.H. (2008). The pdz binding motif of human papillomavirus type 16 e6 induces ptpn13 loss, which allows anchorage-independent growth and synergizes with ras for invasive growth. J. Virol..

[154] Spanos W.C., Geiger J., Anderson M.E., Harris G.F., Bossler A.D., Smith R.B., Klingelhutz A.J., Lee J.H. (2008). Deletion of the pdz motif of hpv16 e6 preventing immortalization and anchorage-independent growth in human tonsil epithelial cells. Head Neck.

[155] Xie L., Yamamoto B., Haoudi A., Semmes O.J., Green P.L. (2006). Pdz binding motif of htlv-1 tax promotes virus-mediated t-cell proliferation *in vitro* and persistence *in vivo*. Blood.

[156] Hirata A., Higuchi M., Niinuma A., Ohashi M., Fukushi M., Oie M., Akiyama T., Tanaka Y., Gejyo F., Fujii M. (2004). Pdz domain-binding motif of human t-cell leukemia virus type 1 tax oncoprotein augments the transforming activity in a rat fibroblast cell line. Virology.

[157] Bartel D.P. (2004). Micrornas: Genomics, biogenesis, mechanism, and function. Cell.

[158] Lewis B.P., Burge C.B., Bartel D.P. (2005). Conserved seed pairing, often flanked by adenosines, indicates that thousands of human genes are microrna targets. Cell.

[159] Calin G.A., Croce C.M. (2006). Microrna signatures in human cancers. Nat. Rev. Cancer.

[160] Lu J., Getz G., Miska E.A., Alvarez-Saavedra E., Lamb J., Peck D., Sweet-Cordero A., Ebert B.L., Mak R.H., Ferrando A.A. (2005). Microrna expression profiles classify human cancers. Nature.

[161] Cai X., Schafer A., Lu S., Bilello J.P., Desrosiers R.C., Edwards R., Raab-Traub N., Cullen B.R. (2006). Epstein-barr virus micrornas are evolutionarily conserved and differentially expressed. PLoS Pathog..

[162] Pfeffer S., Zavolan M., Grasser F.A., Chien M., Russo J.J., Ju J., John B., Enright A.J., Marks D., Sander C. (2004). Identification of virus-encoded micrornas. Science.

[163] Choy E.Y., Siu K.L., Kok K.H., Lung R.W., Tsang C.M., To K.F., Kwong D.L., Tsao S.W., Jin D.Y. (2008). An epstein-barr virus-encoded microrna targets puma to promote host cell survival. J. Exp. Med..

[164] Dolken L., Malterer G., Erhard F., Kothe S., Friedel C.C., Suffert G., Marcinowski L., Motsch N., Barth S., Beitzinger M. (2010). Systematic analysis of viral and cellular microrna targets in cells latently infected with human gamma-herpesviruses by risc immunoprecipitation assay. Cell Host Microbe.

[165] Marquitz A.R., Mathur A., Nam C.S., Raab-Traub N. (2011). The epstein-barr virus bart micrornas target the pro-apoptotic protein bim. Virology.

[166] Marquitz A.R., Raab-Traub N. (2012). The role of mirnas and ebv barts in npc. Semin. Cancer Biol..

[167] Marquitz A.R., Mathur A., Shair K.H., Raab-Traub N. (2012). Infection of epstein-barr virus in a gastric carcinoma cell line induces anchorage independence and global changes in gene expression. Proc. Natl. Acad. Sci. USA.

[168] Blattner W.A. (1999). Human retroviruses: Their role in cancer. Proc. Assoc. Am. Phys..

[169] Kim H.H., van den Heuvel A.P., Schmidt J.W., Ross S.R. (2011). Novel common integration sites targeted by mouse mammary tumor virus insertion in mammary tumors have oncogenic activity. PLoS One.

[170] Sourvinos G., Tsatsanis C., Spandidos D.A. (2000). Mechanisms of retrovirus-induced oncogenesis. Folia Biol..

[171] Chimal-Ramirez G.K., Espinoza-Sanchez N.A., Fuentes-Panana E.M. (2013). Protumor activities of the immune response: Insights in the mechanisms of immunological shift, oncotraining, and oncopromotion. J. Oncol..

[172] Elinav E., Nowarski R., Thaiss C.A., Hu B., Jin C., Flavell R.A. (2013). Inflammation-induced cancer: Crosstalk between tumours, immune cells and microorganisms. Nat. Rev. Cancer.

[173] (1994). Schistosomes, Liver Flukes and Helicobacter Pylori. Iarc working group on the evaluation of carcinogenic risks to humans. Lyon, 7–14 June 1994. IARC Monogr. Eval. Carcinog. Risks Hum..

[174] Fuentes-Panana E., Camorlinga-Ponce M., Maldonado-Bernal C. (2009). Infection, inflammation and gastric cancer. Salud Publica Mex..

[175] Kusters J.G., van Vliet A.H., Kuipers E.J. (2006). Pathogenesis of *Helicobacter pylori* infection. Clin. Microbiol. Rev..

[176] Xu J., Yin Z., Cao S., Gao W., Liu L., Yin Y., Liu P., Shu Y. (2013). Systematic review and meta-analysis on the association between il-1b polymorphisms and cancer risk. PLoS One.

[177] Xue H., Lin B., Ni P., Xu H., Huang G. (2010). Interleukin-1b and interleukin-1 rn polymorphisms and gastric carcinoma risk: A meta-analysis. J. Gastroenterol. Hepatol..

[178] Crusius J.B., Canzian F., Capella G., Pena A.S., Pera G., Sala N., Agudo A., Rico F., Del Giudice G., Palli D. (2008). Cytokine gene polymorphisms and the risk of adenocarcinoma of the stomach in the european prospective investigation into cancer and nutrition (epic-eurgast). Ann. Oncol..

[179] Cheng D., Hao Y., Zhou W., Ma Y. (2013). Positive association between interleukin-8 -251a>t polymorphism and susceptibility to gastric carcinogenesis: A meta-analysis. Cancer Cell Int..

[180] Poli G. (2000). Pathogenesis of liver fibrosis: Role of oxidative stress. Mol. Asp. Med..

[181] Cardenas-Mondragon M.G., Carreon-Talavera R., Camorlinga-Ponce M., Gomez-Delgado A., Torres J., Fuentes-Panana E.M. (2013). Epstein barr virus and helicobacter pylori co-infection are positively associated with severe gastritis in pediatric patients. PLoS One.

[182] Cárdenas-Mondragón M., Carreón-Talavera R., Flores-Luna L., Camorlinga-Ponce M., Gómez-Delgado A., Torres J., Fuentes-Pananá E. Epstein barr virus reactivation is an important trigger of gastric inflammation and progression to intestinal type gastric cancer. Proceedings of the Epstein Barr Virus 50th Anniversary Conference.

[183] Bernstein W.B., Little R.F., Wilson W.H., Yarchoan R. (2006). Acquired immunodeficiency syndrome-related malignancies in the era of highly active antiretroviral therapy. Int. J. Hematol..

[184] Young L.S., Murray P.G. (2003). Epstein-barr virus and oncogenesis: From latent genes to tumours. Oncogene.

[185] Young L., Alfieri C., Hennessy K., Evans H., O’Hara C., Anderson K.C., Ritz J., Shapiro R.S., Rickinson A., Kieff E. (1989). Expression of epstein-barr virus transformation-associated genes in tissues of patients with ebv lymphoproliferative disease. N. Engl. J. Med..

[186] Nourse J.P., Jones K., Gandhi M.K. (2011). Epstein-barr virus-related post-transplant lymphoproliferative disorders: Pathogenetic insights for targeted therapy. Am. J. Transplant..

[187] Gandhi M.K., Wilkie G.M., Dua U., Mollee P.N., Grimmett K., Williams T., Whitaker N., Gill D., Crawford D.H. (2007). Immunity, homing and efficacy of allogeneic adoptive immunotherapy for posttransplant lymphoproliferative disorders. Am. J. Transplant..

[188] Haque T., Wilkie G.M., Jones M.M., Higgins C.D., Urquhart G., Wingate P., Burns D., McAulay K., Turner M., Bellamy C. (2007). Allogeneic cytotoxic t-cell therapy for ebv-positive posttransplantation lymphoproliferative disease: Results of a phase 2 multicenter clinical trial. Blood.

[189] Michaelis M., Doerr H.W., Cinatl J. (2009). The story of human cytomegalovirus and cancer: Increasing evidence and open questions. Neoplasia.

[190] Boldogh I., Huang E.S., Rady P., Arany I., Tyring S., Albrecht T. (1994). Alteration in the coding potential and expression of h-ras in human cytomegalovirus-transformed cells. Intervirology.

[191] Geder K.M., Lausch R., O’Neill F., Rapp F. (1976). Oncogenic transformation of human embryo lung cells by human cytomegalovirus. Science.

[192] Geder L., Kreider J., Rapp F. (1977). Human cells transformed *in vitro* by human cytomegalovirus: Tumorigenicity in athymic nude mice. J. Natl. Cancer Inst..

[193] Geder L., Laychock A.M., Gorodecki J., Rapp F. (1978). Alterations in biological properties of different lines of cytomegalorivus-transformed human embryo lung cells following *in vitro* cultivation. IARC Sci. Publ..

[194] Cinatl J., Scholz M., Kotchetkov R., Vogel J.U., Doerr H.W. (2004). Molecular mechanisms of the modulatory effects of hcmv infection in tumor cell biology. Trends Mol. Med..

[195] Scheurer M.E., Bondy M.L., Aldape K.D., Albrecht T., El-Zein R. (2008). Detection of human cytomegalovirus in different histological types of gliomas. Acta Neuropathol..

[196] Cobbs C.S., Soroceanu L., Denham S., Zhang W., Kraus M.H. (2008). Modulation of oncogenic phenotype in human glioma cells by cytomegalovirus ie1-mediated mitogenicity. Cancer Res..

[197] Maussang D., Verzijl D., van Walsum M., Leurs R., Holl J., Pleskoff O., Michel D., van Dongen G.A., Smit M.J. (2006). Human cytomegalovirus-encoded chemokine receptor us28 promotes tumorigenesis. Proc. Natl. Acad. Sci. USA.

[198] Vallat L., Benhamou Y., Gutierrez M., Ghillani P., Hercher C., Thibault V., Charlotte F., Piette J.C., Poynard T., Merle-Beral H. (2004). Clonal b cell populations in the blood and liver of patients with chronic hepatitis c virus infection. Arthritis Rheum..

[199] Quinn E.R., Chan C.H., Hadlock K.G., Foung S.K., Flint M., Levy S. (2001). The b-cell receptor of a hepatitis c virus (hcv)-associated non-hodgkin lymphoma binds the viral e2 envelope protein, implicating hcv in lymphomagenesis. Blood.

[200] Chan C.H., Hadlock K.G., Foung S.K., Levy S. (2001). V(h)1–69 gene is preferentially used by hepatitis c virus-associated b cell lymphomas and by normal b cells responding to the e2 viral antigen. Blood.

[201] Hermine O., Lefrere F., Bronowicki J.P., Mariette X., Jondeau K., Eclache-Saudreau V., Delmas B., Valensi F., Cacoub P., Brechot C. (2002). Regression of splenic lymphoma with villous lymphocytes after treatment of hepatitis c virus infection. N. Engl. J. Med..

[202] Zullo A., Hassan C., Cristofari F., Andriani A., de Francesco V., Ierardi E., Tomao S., Stolte M., Morini S., Vaira D. (2010). Effects of helicobacter pylori eradication on early stage gastric mucosa-associated lymphoid tissue lymphoma. Clin. Gastroenterol. Hepatol..

[203] Skinner G.R. (1976). Transformation of primary hamster embryo fibroblasts by type 2 simplex virus: Evidence for a “hit and run” mechanism. Br. J. Exp. Pathol..

[204] Birdwell C.E., Queen K.J., Kilgore P.C., Rollyson P., Trutschl M., Cvek U., Scott R.S. (2014). Genome-wide DNA methylation as an epigenetic consequence of epstein-barr virus infection of immortalized keratinocytes. J. Virol..

[205] Burns M.B., Temiz N.A., Harris R.S. (2013). Evidence for apobec3b mutagenesis in multiple human cancers. Nat. Genet..

